# Ten simple rules for managing communications with a large number of coauthors

**DOI:** 10.1371/journal.pcbi.1010185

**Published:** 2022-06-30

**Authors:** Robert Muscarella, Lourens Poorter

**Affiliations:** 1 Department of Ecology and Genetics, Plant Ecology and Evolution, Uppsala University, Uppsala, Sweden; 2 Forest Ecology and Forest Management Group, Wageningen University & Research, Wageningen, the Netherlands; Dassault Systemes BIOVIA, UNITED STATES

## Introduction

The grand challenges of the 21st century, such as global change, biodiversity loss, and sustainable resource use, can only adequately be addressed through global collaboration and global solutions. Such solutions are ideally evidence based and require a mechanistic understanding of the underlying drivers and processes operating at different spatial–temporal scales. This requires the synthesis and analysis of large datasets to draw strong generalizations, while at the same time appreciating the context dependence and local deviations from these generalizations. Such cutting-edge advances in theoretical and applied research are therefore increasingly based on large collaborations [1]. Big research teams can integrate large and heterogeneous data sources, leverage a broad range of technical expertise, provide detailed insights in the local reality of different study systems, and wield advanced skills in statistics, computer programming, and interpretation. During the past decades, these factors have led to a substantial increase in the number of coauthors on scientific papers [2,3]. However, effectively communicating with a large number of coauthors presents multiple challenges.

The objective of this guide is to provide recommendations for effective communication with large coauthor teams. The recommendations can be helpful for manuscripts involving relative few authors (approximately 10), but the benefits will increase as the size of your coauthor team grows to dozens or hundreds of coauthors. These “rules” are based on lessons learned from personal experience [4–6]. Of course, alternatives exist, but we hope that our suggestions will help to promote an efficient and equitable writing process.

Numerous software applications have been developed to aid collaborative work (try googling “collaborative project software”). If you and your coauthors are already comfortable using one or more of these platforms (congratulations!), then some of our suggestions may seem obvious or irrelevant. However, many people may be unwilling or unable to engage with these specialized platforms because of a diversity of cultures, regions, backgrounds, or research fields among coauthors. This guide will be most valuable for lead author(s) of large coauthor teams for whom using specialized collaborative software is unrealistic or undesirable.

Several important issues for large coauthor groups are not addressed by this guide, such as how to determine eligibility for coauthorship or author order. Journals typically publish their standards for coauthorship (e.g., https://journals.plos.org/ploscompbiol/s/authorship), and many online resources can be found to guide the assessment what contributions should warrant coauthorship [7]. This guide neither focuses on the actual writing process (e.g., size and structure of the core lead writing team and division of labor), considerations which are addressed by an existing “10 simple rules” paper [8]. Finally, in a broad sense, successful collaborations require a commitment to an inclusive, respectful, and trusting atmosphere; the advice offered here is only a small part of this much larger conversation [9,10]. We hope the suggestions offered below will help reduce barriers to efficient and productive communication with your (many!) coauthors.

### Rule 1: Establish communication as a priority

The importance of establishing communication as a priority among your coauthors cannot be overstated. As a lead author(s) on a many-authored paper, logistic and other challenges may tempt you to write the manuscript in complete isolation and then send a nearly final version out for final (minor) comments. Besides the fact that this approach runs counter to most guides that define appropriate authorship contribution [11], this (anti-) strategy also ensures that you will alienate at least some of your coauthors, and it actively silences the diverse perspectives that are a major strength of your coauthor team. Establishing an atmosphere that encourages communication and feedback is essential for bringing the voices and ideas of many into your final manuscript [9]. Moreover, having well-established channels of communication will help facilitate resolutions when there are complications or conflicts among coauthors. One good way to promote communication in your project is to discuss preliminary results during videoconference meetings (with up to 20 people), with specific questions to be discussed in breakout groups who then report back to whole group. If more people want to join, organize several meetings in the same week, which can also accommodate different time zones. Overall, be sure to start your project on the right foot by explicitly discussing your approach for communication with your coauthors.

### Rule 2: Create a spreadsheet to track coauthor details

Now on to nuts and bolts. Organizing all those names and affiliations can be a major pain, but life will be much easier if you treat this information as other precious data: Keep it well-organized in a machine-readable spreadsheet (e.g., S1 Table). Make separate columns for first and last names and initials (and remember that many people have more than one first and last names). Ensure that your spreadsheet uses text encoding that does not garble accented or other characters. Keeping separate columns for affiliation details such as “country,” “institute,” and “address” will help you identify duplicate affiliations that people may have written down slightly differently (this helps keep affiliation numbering consistent). When preparing your manuscript, you can also use this spreadsheet to automate the process of generating the author list (including numbered affiliations) by using a programming language (see more on this below).

You can also use the author spreadsheet to list the author contributions (data provisioning, analysis, core writing team, and providing comments), which may be required by the journal. It is also very convenient to track if and when each coauthor provides comments (and send them a reminder when they have not done so) as we as when they give their final approval for manuscript submission. Finally, documentation on funding sources, competing interests, or other such statements can also be included to facilitate administrative work done by the lead author(s). To save time and ensure accuracy, it may be useful to have coauthors fill out part of these data using a standardized online form (e.g., Google Forms).

### Rule 3: Prepare your email system

Attempting to manage communications for a many-authored paper using your primary email address can quickly lead to inbox overflow and wild confusion. At best, this is annoying for keeping your inbox organized. At worst, it can negatively impact your other responsibilities and mental well-being. There are numerous approaches for avoiding this disaster. One option is to set up a separate email account devoted specifically to the project. This can help give the project its own identity (both for you and for your coauthors), and it allows you to isolate the project from the rest of your work. If it makes sense in your case, a separate email account also allows you to share access with key collaborators as opposed to forwarding piles of emails from your personal account. In our experience, setting up a separate (free) Gmail account works well for several reasons, elaborated below. Note that multiple Gmail accounts can be set to feed into a single inbox so having multiple addresses does not require multiple sign-ins or separate tabs open in your browser. You may, however, prefer using tags and filtering options with your normal email address to segregate messages. Gmail has a helpful guide for setting up these types of options (https://support.google.com/a/users/answer/9282966?hl=en). In any case, the key message here is to prepare your email system in advance to avoid chaos.

### Rule 4: Send individual (not group) emails

Even with the most generic communications about the project, we highly recommend to avoid sending group emails (e.g., with all coauthors in BCC or—heaven forbid!—in normal CC) and instead send separate emails to each individual coauthor. Why? Because otherwise you will end up with a massive email chain with hundreds of reply emails from separate people. These chains are extremely difficult to search and keep up with. It is much (much!) easier to have separate email threads for each individual person. That way you can easily track and search communications with individual people as needed. You end up with, say, 100 separate email threads in your inbox rather than an unwieldy medusa thread of 100 emails from 100 people that continues to grow in all directions. Importantly, from the perspective of your coauthors, an individual email will appear identical to a group email with people in BCC. But, from your side, managing the various conversations with individual coauthors is much more manageable, and it also opens the opportunity to personalize your messages (more on this below).

### Rule 5: Learn (or collaborate with someone with) basic programming skills

Computers are designed to help us (right?), and the following 2 rules involve using basic computer programming to efficiently communicate with your army of coauthors. While some (if not all) of the suggestions below can also be achieved using more specialized software designed for collaborative projects, we focus on an approach that does not require your coauthors to engage with any additional platform beyond email. The below steps can be achieved with a basic knowledge of various programming languages but, in our experience, the (free) R software environment [12] works very well for these tasks. For intermediate to seasoned programmers, the suggestions below will present no real obstacle. For those who may be timid about working with a command line program, you will want to take some time to learn the basics or find a collaborator who can help you navigate this process. Fortunately, there are many good beginners guides available (e.g., [13]) and with a big list of coauthors, you are likely to have at least one who can help.

### Rule 6: Automate the process of sending individual emails

For the individual email approach to be practical, you will need to automate the process. For some, this may sound like computer science sorcery but it really can be simple. In some cases, you may want to add other pertinent personalized information (preferred language, previous response to a question, etc.) to the author data spreadsheet (see above). You can then draft generic email text, which you later concatenate with the name of the person (for some personalization) using R. You may also find it useful to split authors into groups with semigeneric text relevant to their subgroup. For example, for multilingual author groups, you could draft your email in different languages and have a “language_ID” column in your spreadsheet to select which language to use for different people.

If you set up a Gmail account for your project and are using R to automate your process, we recommend the “gmailr” R package [14], which provides a nice tutorial for an overview of the process (https://gmailr.r-lib.org). There are other R packages for emailing, including “emayili” [15], which may work well depending on the specifics of your email setup. In any case, you will definitely want to get familiar with an automated email tool that works for you.

### Rule 7: Test your automated emailing system

One thing you do not want to do is send hundreds of automated emails with incorrect information, typos, or missing attachments. To avoid these headaches, you should always thoroughly test your automated email system by creating drafts before sending, at least for a subset of authors. Inspect the drafts to ensure that everything is as expected before going ahead with “the big send.” We also often use the automated system to send emails to ourselves and confirm things are working as expected. Generating the draft emails and, ultimately, sending the many individual emails to each coauthor can be achieved in an automated way using a basic loop in your program script (see R code in S1 Data).

### Rule 8: Send PDFs of draft manuscripts and request feedback in a spreadsheet form

**Fig 1:**
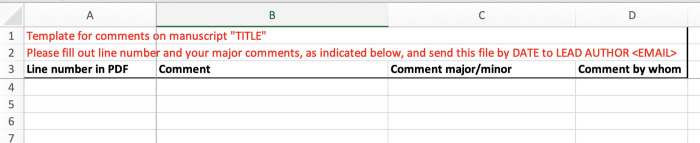
Example of spreadsheet template for soliciting comments from coauthors. Once filled, the spreadsheets from multiple coauthors can be combined and sorted by line number. This file is provided as S2 Table.

Now that you have built a system for contacting your coauthors, you are ready to solicit comments on your draft manuscript. In our experience, the most common way that people request feedback from coauthors is by using the Track Changes feature of Microsoft Word. Even in the best-case scenario, using Track Changes can be exhausting (with many coauthors, it is completely untenable). Writing a collaborative document with a shared platform (e.g., Google Docs) may offer some advantages, but you will sooner or later still be confronted by a psychedelic rainbow of overlapping comments that become unreadable. Moreover, people have different writing styles and often contrasting views, so you can quickly lose control over the direction of the manuscript and the coauthors have lost the overview of the contents. In our opinion, the best solution is to send a line-numbered manuscript draft as a PDF together with a preformatted spreadsheet (Fig 1) where people can add the line number, their comments, and indicate whether it is a major or minor comment. You may want to explicitly tell coauthors that you only consider comments added to the spreadsheet template because some people prefer to comment directly on the PDF, which puts the burden on you as lead authors to compile all comments and keep the overview. Typing comments in a spreadsheet also assures that your coauthors focus on major comments rather than on minor phrasing issues and micro-edits. Once people return the filled comment spreadsheets, you can again use a programming software to merge the rows from different sheets and save a single file with all comments ordered by line number. From here, it will be relatively easy to condense the dozens of comments about, for example, a single typo. You will also easily be able to reach out to individual coauthors, for example, for clarification on a specific comment. And the real power is that for the same line/paragraph you at once see 10 different suggestions how to deal with the issue, and pick out the best. This is where you really can benefit from the strength of the different perspectives, arguments, and phrasing of your coauthor team.

After the core writing team has addressed all the comments, you should send the revised version of the manuscript back to all coauthors with an explanation of how you dealt with the comments. If you are brave, you can attach the Excel sheet with a column added next to each remark about how it was (or wasn’t) dealt with. This way people can see comments from other coauthors and how you dealt with the suggestions. Otherwise, your coauthors may understand why you sometimes did not include their comments, or why you did it differently, and they may feel like they are not being taken seriously.

### Rule 9: Be clear about your process and deadlines

Waiting for comments from a single coauthor can be frustrating but, if you get desperate, you can usually pick up the phone or send a quick message to remind them about a deadline. With many coauthors, it is critical to include concrete details about your process—including expectations and deadlines—in your communications and that comments after the deadline are not included. Of course, you could provide extensions if there are good reasons but setting clear deadlines and sending reminders to people who have not yet responded will make your life easier. Expectation management is also important. Do you expect for this manuscript version mainly ideas, major comments, detailed comments, or—when you are close to submission—only a last check for major issues and factual errors? Giving a deadline of, say, 3 weeks is often OK, but take into account the holiday seasons in different regions, if you have a global distribution of coauthors. If you have a tight planning, you can also ask coauthors a few weeks in advance to block their agenda and assure a fast reply when you send the manuscript.

When specific tasks (e.g., comments on a draft) are required from your coauthors, it is useful to add a column in your coauthor tracking spreadsheet (Rule 2) to note if and when each person has responded or completed the task. This will enable you to easily use your automated email system to easily reach out and nudge people who have not yet completed the task.

### Rule 10: Provide opportunities for discussion outside of email

Arguably, a major “silver lining” of the Coronavirus Disease 2019 (COVID-19) pandemic is the general increase in our ability to remotely connect with others. Large coauthor groups are typically spread across the globe and rarely have opportunities to interact in person. Moreover, many of your coauthors may not know one another but they clearly have shared interests. Creating opportunities during the writing process to meet online and discuss the work (as well as socialize and network, in general) can be a great way to get more feedback and strengthen the group. Who knows? It might even lead to another many-authored paper.

## Conclusions

Research projects involving large groups typically lead to publications with many coauthors. While this is an important outcome to credit people for their contributions, it can involve substantial logistic challenges for communication. Hopefully, the pragmatic suggestions in this guide help make communication more efficient and, ultimately, facilitate a more productive, trusting, and fair writing process.

## Supporting information

S1 Data Example R script. (R)

S1 Table Coauthor spreadsheet. (CSV)

S2 Table Template for comments. (XLSX)
